# The Influence of Verbatim Versus Gist Formatting on Younger and Older Adults’ Information Acquisition

**DOI:** 10.1093/geroni/igaa057.1831

**Published:** 2020-12-16

**Authors:** Julia Nolte, Corinna Loeckenhoff, Valerie Reyna

**Affiliations:** Cornell University, Ithaca, New York, United States

## Abstract

It is well-established that pre-decisional information seeking decreases with age (Mata & Nunes, 2010). However, it is still unknown whether age differences in information acquisition are influenced by the type of information provided. Fuzzy-trace theory suggests that decision makers prefer gist-based over verbatim-based processing, and that this preference increases across the lifespan. Therefore, we hypothesized that age differences arise when presenting participants with verbatim details (such as exact numbers) but not gist information (such as ”extremely poor” or “good”). In a lab-based experiment, 68 younger adults and 66 older adults completed a gist-based and a verbatim-based search task before making health insurance choices. Younger and older adults reviewed similar amounts of information in either condition. In line with Fuzzy-trace theory, however, older adults sought more information when presented with gist rather than verbatim information. The role of age-associated covariates and implications for decision-making will be discussed.

